# pH homeostasis in yeast; the phosphate perspective

**DOI:** 10.1007/s00294-017-0743-2

**Published:** 2017-08-30

**Authors:** Elja Eskes, Marie-Anne Deprez, Tobias Wilms, Joris Winderickx

**Affiliations:** Functional Biology, KU Leuven, Kasteelpark Arenberg 31 box 2433, 3001 Heverlee, Belgium

**Keywords:** PKA, TORC1–Sch9, V-ATPase, Pma1, PHO pathway, Polyphosphates, Inositol pyrophosphates

## Abstract

Recent research further clarified the molecular mechanisms that link nutrient signaling and pH homeostasis with the regulation of growth and survival of the budding yeast *Saccharomyces cerevisiae*. The central nutrient signaling kinases PKA, TORC1, and Sch9 are intimately associated to pH homeostasis, presumably allowing them to concert far-reaching phenotypical repercussions of nutritional cues. To exemplify such repercussions, we briefly describe consequences for phosphate uptake and signaling and outline interactions between phosphate homeostasis and the players involved in intra- and extracellular pH control. Inorganic phosphate uptake, its subcellular distribution, and its conversion into polyphosphates are dependent on the proton gradients created over different membranes. Conversely, polyphosphate metabolism appears to contribute in determining the intracellular pH. Additionally, inositol pyrophosphates are emerging as potent determinants of growth potential, in this way providing feedback from phosphate metabolism onto the central nutrient signaling kinases. All these data point towards the importance of phosphate metabolism in the reciprocal regulation of nutrient signaling and pH homeostasis.

## Introduction

Yeast cells possess a complex network of signal cascades enabling them to make appropriate adjustments in metabolism in response to environmental changes. Here we summarize recent advances in the field with emphasis on the link between nutrient signaling and the regulation of pH homeostasis, thereby highlighting the emerging connections with the uptake and metabolism of inorganic phosphate (Pi).

It is well established that the protein kinases PKA and Sch9, and the Tor Complex 1 (TORC1) play a central role in the nutrient-induced signaling network that controls growth, survival, and longevity by maintaining a tight balance between proliferation and stress defense (Ho and Gasch 2015; Longo et al. 2012; Smets et al. 2010). PKA activity is regulated by the Ras–cAMP pathway and activation of adenylate cyclase. The latter requires extracellular sensing of glucose via the Gpr1–Gpa2 GPCR system as well as intracellular glucose sensing via the hexokinases Hxk1/2 and glucokinase Glk1, which in turn stimulate the small GTPases Ras1 and Ras2 (Colombo et al. 2004; Rolland et al. 2000). Nitrogen sources activate TORC1 at the vacuolar membrane. Depending on their quality as nitrogen source, amino acids act through an evolutionary conserved mechanism comprising EGOC, a complex containing the Rag-like GTPases Gtr1 and Gtr2 (Hatakeyama and De Virgilio 2016; Powis and De Virgilio 2016). Sch9 is a well-known TORC1 effector and shuttles between the cytoplasm and the vacuole in a glucose-dependent manner (Wilms et al. 2017). Besides TORC1, Sch9 activity is also regulated by the sphingolipid-dependent PDK1 orthologues Pkh1-3 and the protein kinase Snf1, a key player in glucose repression (Smets et al. 2010; Swinnen et al. 2014). The pathways controlled by PKA, TORC1, and Sch9 converge on the protein kinase Rim15, which ensures proper entry into the stationary phase by activating the expression of the so-called STRE- and PDS-controlled genes during the diauxic shift (Smets et al. 2010). The resulting metabolic adjustments and increased stress resistance have been shown to be vital for the survival of stationary phase cells (Longo et al. 2012; Lopez-Otin et al. 2016; Pedruzzi et al. 2003). Rim15 also provides a first link to phosphate signaling, since its nuclear exit is under control of the cyclin-kinase complex Pho80–Pho85, a main regulator of the Pi-responsive PHO pathway, as further explained below (Smets et al. 2010). Among the genes controlled by Rim15 and Pho80–Pho85 are those encoding the subunits of the trehalose synthase complex. As such, Pi availability and the activity status of Pho80–Pho85 directly impact on the biosynthesis of trehalose, a disaccharide important for survival during different kinds of stresses, including nutrient starvation. When the stress is halted or when nutrients are again plentiful, trehalose is rapidly degraded and, here, the availability of Pi dictates the PKA-dependent activation of Nth1, the neutral trehalase that hydrolyzes the disaccharide in the cytoplasm (Eleutherio et al. 2015). This simple example already illustrates the close interplay of the nutrient-dependent pathways controlled by PKA, TORC1, and Sch9, and phosphate signaling.

**Fig. 1:**
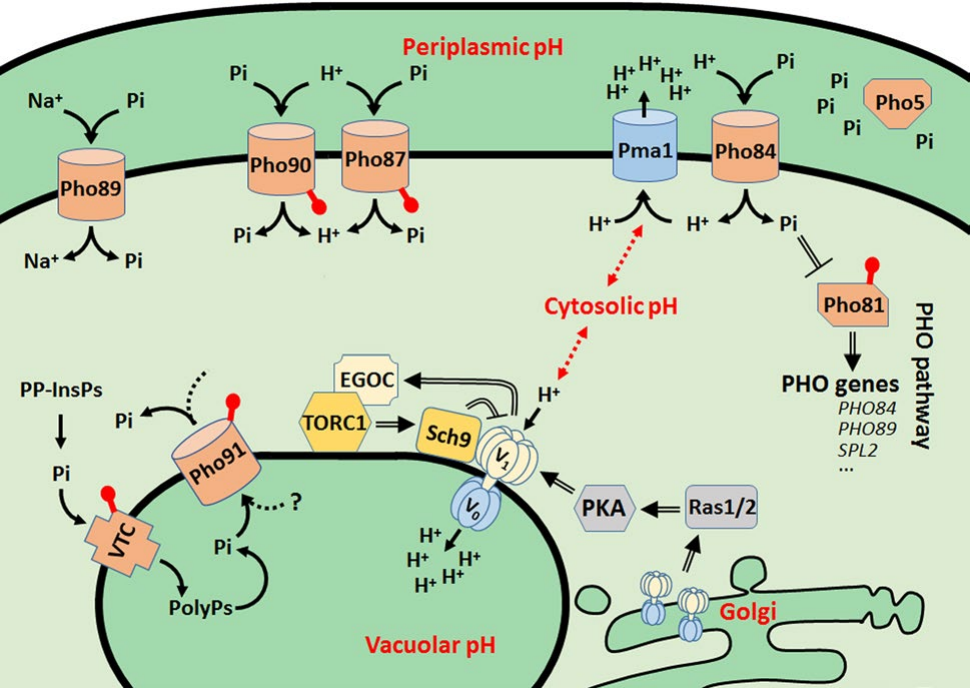
Schematic overview of the players involved in Pi homeostasis, pH homeostasis, and control of the vacuolar V-ATPase assembly/disassembly state in function of glucose availability. *Closed arrows* indicate events involved in Pi homeostasis, *open arrows* to refer to signaling effects. The *red dots* represent the SPX domains. *PolyPs* polyphosphates, *PP-InsPs* inositol pyrophosphates

Recent advances on the control of pH homeostasis revealed interesting new aspects underpinning the cross-talk within the nutrient signaling network. In 2010, the cytosolic proton was proposed to act as bona fide second messenger based on the observation that Ras-GTP load, the activity state of PKA, and the transcription of STRE-dependent genes correlated with glucose-dependent alterations of the cytosolic pH and with the assembly/disassembly of the vacuolar proton pump V-ATPase (Dechant et al. 2010). This (dis)assembly is the main regulatory mode for V-ATPase activity (Kane 2016). A follow-up study then identified the small GTPases Arf1 and Gtr1 as the V-ATPase interactors that transmit a signal of active V-ATPases in the Golgi and vacuole towards Ras–PKA and TORC1, respectively (Dechant et al. 2014). Most recently, we further clarified the mutual interplay of the players mentioned above, as we found that deletion of the TORC1-effector *SCH9* retards the disassembly of the V-ATPase upon glucose starvation, resulting in reduced vacuolar pH (Wilms et al. 2017). A similar effect was reported for mutants with enhanced PKA activity (Bond and Forgac 2008), indicating opposite effects of PKA and TORC1/Sch9 on V-ATPase disassembly, as depicted in Fig. 1. In addition, we demonstrated that the cytosolic pH of *sch9Δ* cells becomes more acidic than that of wild-type cells when these traverse the diauxic shift. This effect was also related to a reduced activity of Pma1, the plasma membrane H^+^-ATPase responsible for proton extrusion, because both the *sch9Δ* mutant and V-ATPase defective mutant display hampered proton efflux, when glucose is supplemented to glucose-deprived cells (Wilms et al. 2017). This proton efflux is essential to sustain the proton gradient across the plasma membrane, which in turn is the driving force for the uptake and extrusion of a wide array of compounds through proton-dependent co-transporters, including Pi acquisition by Pi symporters. As shown in Fig. 1, yeast cells possess two low-affinity H^+^/Pi symporters, Pho87 and Pho90, and one high-affinity H^+^/Pi symporter, Pho84. In addition, they also have a high-affinity Na^+^/Pi symporter, Pho89, and a low-affinity phosphate transporter localized in the vacuole membrane, Pho91, which is essential for the storage and mobilization of polyphosphates that serve as Pi reserve (Hurlimann et al. 2007; Persson et al. 2003). The Pi symporters do not affect the cytosolic or vacuolar pH during fermentative growth (Brett et al. 2011; Orij et al. 2012). This is expected since Pma1 is highly active under these conditions and immediately compensates by proton efflux. However, whether Pi symporters, and especially Pho84, contribute to pH homeostasis under post-diauxic shift conditions with reduced Pma1 activity (Kane 2016) has to our knowledge not been investigated.

Pho84 is responsible for most of the Pi uptake in yeast, even under plentiful growth conditions (Ghillebert et al. 2011; Persson et al. 2003; Wykoff and O’Shea 2001). Consistent with the fact that this symporter is sensitive to changes in the extracellular and cytosolic pH is the observation that high-affinity Pi transport is compromised in a partial loss-of-function *pma1* mutant, and that this can be restored by lowering the extracellular pH (Lau et al. 1998). Furthermore, loss of the proton gradient was shown to be sufficient to mimic a phosphate-limitation response leading to activation of the PHO pathway (Lau et al. 1998; Serrano et al. 2002). In this pathway, the CDK-inhibitor Pho81 acts as gatekeeper. Its activation relieves the inhibitory effect of Pho80–Pho85 on the Pho4 transcription factor, which together with its co-activator Pho2 controls expression of genes required for phosphate acquisition. These genes include the high-affinity symporters Pho84 and Pho89, the acidic phosphatase Pho5, and Spl2, a protein necessary for downregulation and vacuolar targeting of the low-affinity transporter Pho87 (Ghillebert et al. 2011; Smets et al. 2010). Also several other genes are upregulated upon the activation of Pho4 (Ogawa et al. 2000; Zhou and O’Shea 2011). These include genes encoding subunits of the vacuolar transporter chaperone (VTC) complex, which are important for the synthesis of polyphosphates (PolyPs) (Ogawa et al. 2000), as well as the gene encoding the phosphatase Phm8 that allows cells to acquire phosphate through the hydrolysis of lysophosphatidic acid and nucleotide monophosphates, and that has an important role in lipid biosynthesis as well (Yadav et al. 2016).

Being such an important symporter, the activity of Pho84 is tightly controlled. Pho84 reaches its highest levels at mid-exponential growth under phosphate-limitation conditions, and afterwards the symporter is gradually internalized and sorted to the vacuole (Lagerstedt et al. 2002). The degradation of Pho84 in these phosphate-limited cells is dramatically enhanced upon phosphate addition. Interestingly, this response is significantly delayed by the inhibition of PKA, which is nicely paralleled by maintenance of high-affinity Pi uptake (Mouillon and Persson 2005). Two other studies also provided a link to TORC1 through the EGOC subunit Gtr1. They showed that cells harboring a deletion of *PHO84* or *GTR1* share similar phenotypes and that *gtr1Δ* cells grown in phosphate-limiting conditions are marked by a delayed derepression of Pho84, as shown by a longer time to reach the maximal Pi uptake and in turn a somewhat belated Pho84 internalization. Notably, the lack of Gtr1 also results in delayed derepression of the acidic phosphatase Pho5 under phosphate limitation. For the fast internalization of Pho84 triggered by phosphate resupplementation, however, the *gtr1Δ* cells behave similar as wild-type cells (Bun-Ya et al. 1992; Lagerstedt et al. 2005). Thus, as is the case for V-ATPase regulation, PKA and TORC1 appear to exert complementary effects on Pho84-dependent Pi uptake, leading to the question of how far this relates to changes in cellular pH homeostasis. To our knowledge, this aspect has not been investigated thoroughly but studies on the life cycle of Pho84 made some interesting observations. A first study demonstrated that Pho84 maintains an optimal activity at an extracellular pH between 5.5 and 7, and once above a pH of 7.5, its activity rapidly declines (Lagerstedt et al. 2002). This study actually aimed to decipher the importance of a Pho84 C-terminal domain sharing homology to a domain of the pheromone receptor Ste2 that determines when this receptor is endocytosed. The deletion of this domain in Pho84 does not influence its endocytosis. However, it significantly reduces the maximal Pi-uptake capacity, renders the symporter very sensitive to protonophore treatment, and extends the pH optimum for Pi uptake towards a more acidic environment, ranging now from pH 4.5 to pH 7 (Lagerstedt et al. 2002). Thus, this domain could have a role in stabilizing Pho84 in function of pH. A second study dealt with the rapid Pi-induced endocytosis of Pho84 and revealed that PKA does not seem to target the symporter. The kinase rather enhances the efficacy of the endocytic process by interfering with the vacuolar protein sorting/multivesicular body (MVB) pathway, which is required to correctly deliver the symporter to the vacuole (Lundh et al. 2009). The same study reported that proper ubiquitination is also essential for the delivery of Pho84 to the plasma membrane (Lundh et al. 2009). This suggests that Pho84 is subject to intracellular sorting and trafficking control (Hicke and Dunn 2003; Kriel et al. 2011), a process known to be affected by starvation-induced TORC1 inactivation and shown to deviate ubiquitinated plasma membrane proteins to the MVB pathway for subsequent vacuolar degradation (Dobzinski et al. 2015). This probably explains the delayed Pho84 derepression observed in the *gtr1Δ* mutant (Lagerstedt et al. 2005). More important, however, is that the presumed roles of PKA and TORC1 in Pma1 regulation lie in the sorting of endocytosed, recycled, and newly synthesized proteins, and indeed, this sorting is highly dependent on the functioning of the vacuolar V-ATPase (Li and Kane 2009).

Apart from being important for proper sorting and degradation of the Pi symporters, the V-ATPase controls another important aspect of phosphate homeostasis. In the vacuole and ER, phosphate is stored in the form of PolyPs, which do not only serve as reserve in times of Pi scarcity, but have also been suggested to contribute in determining the final pH of the organelles (Li and Kane 2009). As mentioned, PolyPs are synthesized by the VTC complex (Ogawa et al. 2000). This complex consists of different subunits that form a channel coupling the synthesis of PolyPs to their translocation across the membrane. The VTC complex was initially found to be required for sorting and stability of the vacuolar V-ATPase, for the correct delivery and distribution of Pma1p in the plasma membrane, for microautophagy as well as for homotypic vacuolar fusion (Cohen et al. 1999; Muller et al. 2002, 2003; Uttenweiler et al. 2007). In many of these processes either PKA, the TORC1–Sch9 axis or both have been implicated (Bond and Forgac 2008; Cebollero and Reggiori 2009; Stauffer and Powers 2015; Wilms et al. 2017), indicating a close link between the activity of the VTC complex and the kinases controlling and being controlled by the V-ATPase. Moreover, an older study showed that ammonium salts, potent activators of TORC1–Sch9 (Stracka et al. 2014), reduce the PolyP content in the vacuole and increase Pi level in the cytoplasm, when supplied to stationary phase cells (Greenfield et al. 1987).

The synthesis of PolyPs is strictly dependent on the proton gradient created by the V-ATPase, as this drives the coupled synthesis–translocation activity of the VTC complex. Consistently, the synthesis of long-chain PolyPs is compromised, when the activity of the V-ATPase is pharmacologically inhibited and in cells lacking the V-ATPase subunit Vma2. In addition, the total PolyP content and chain length in wild-type cells not only depend on Pi supply, but also differ significantly depending on the growth phase and availability of either ethanol or glucose as carbon source (Tomaschevsky et al. 2010; Trilisenko et al. 2013). Given the compensatory interplay between the V-ATPase and Pma1, it is not surprising that mutations in Pma1 also affect the chain length of PolyPs (Tomashevski and Petrov 2015a, b).

Pi starvation enhances the expression of the endopolyphosphatase Ppn1 that hydrolyzes PolyPs in the vacuole (Gerasimaite and Mayer 2016). Once PolyPs are broken down by Ppn1, the hydrolyzed Pi is exported from the vacuole into the cytosol presumably by the low-affinity transporter Pho91 (Hurlimann et al. 2007; Yang et al. 2017). To this date, it remains unclear whether this transporter acts as proton-driven symporter. However, similar as Vtc2, Vtc3, and Vtc4, the Pho91 transporter contains a SPX domain. These domains have a role in protein interaction and function as sensors for inositol pyrophosphates (PP-InsPs) (Gerasimaite et al. 2017; Secco et al. 2012; Wild et al. 2016). Indeed, PP-InsPs have been shown to regulate PolyP metabolism, as evidenced by the observations that mutants lacking either Plc1, Arg82 or Kcs1, enzymes involved in PP-InsP biosynthesis, show significantly reduced levels or even completely lack PolyPs (Auesukaree et al. 2005; Lonetti et al. 2011). Interestingly, the *plc1Δ* and *kcs1Δ* mutants are also characterized by a lower cytosolic pH (Orij et al. 2012), suggesting that an altered proton gradient over the vacuolar membrane may be the reason for their abrogated PolyP synthesis. The SPX domain is also present in other proteins involved in Pi homeostasis, like Pho81 and the H^+^/Pi symporters Pho87 and Pho90, thereby controlling important aspects of their functioning (Secco et al. 2012). For instance, the SPX domain controls the Pi starvation-induced endocytosis of Pho90 and Pho87 (Ghillebert et al. 2011; Hurlimann et al. 2009). Interestingly, the SPX domain also drives the endocytosis of these symporters upon glucose starvation and rapamycin treatment (Ghillebert et al. 2011), again providing a link to PKA and TORC1–Sch9. Finally, PP-InsPs have been reported to control the activation of the PHO pathway in concert with Pi availability by allosterically regulating the Pho81–Pho80–Pho85 interaction. This regulation requires the so-called minimum domain in the C terminus of Pho81, but here the SPX domain seems not to be essential. (Auesukaree et al. 2005; Lee et al. 2007, 2008). Importantly, for the regulation of stress responses by Pho80–Pho85 and Rim15, the minimum domain is not sufficient but depends on other regions in Pho81 as well (Swinnen et al. 2005).

Mutants lacking Plc1 or Kcs1 are among the few strains that fail to adjust growth rate in function of cytosolic pH (Orij et al. 2012). The underlying reasons can be diverse but it certainly reflects the central role played by PP-InsPs in the overall cellular homeostasis (Wundenberg and Mayr 2012). For instance, PP-InsPs fine-tune inositol metabolism via Kcs1 through *INO1* transcription and interplay with Opi1, a transcriptional repressor known to be regulated by glucose and intracellular pH, thereby linking nutrient availability to membrane biogenesis (Ye et al. 2013; Young et al. 2010). Intriguingly, Sch9 was found to genetically interact with *KCS1*, *OPI1*, *INO1*, and other players involved in inositol metabolism (Wilms et al. 2017), which likely relates to the fact that PI[3,5]P_2_ is required for the recruitment of Sch9 to the vacuolar membrane and its subsequent TORC1-dependent phosphorylation (Jin et al. 2014, 2016; Jin and Weisman 2015). PP-InsP metabolism also connects to nutritional stress management as *plc1Δ* cells display enhanced Msn2-mediated transcription of STRE-genes, decreased cAMP levels, and reduced PKA activity (Demczuk et al. 2008), the latter being consistent with Plc1 mediating the interaction of Gpr1 with Gpa2, constituting the GPCR system needed for extracellular glucose sensing (Ansari et al. 1999). Furthermore, similar to *plc1Δ* cells, the mutant lacking Gpr1 displays reduced levels of IP_3_, a precursor for PP-InsP synthesis (Demczuk et al. 2008). How the role of Plc1 relates to the Pi-induced activation of trehalase as triggered by the symporters Pho87- and Pho84-mediated (Giots et al. 2003) remains to be analyzed, but certain is that this activation requires cells to be pre-grown and starved for Pi on a glucose-containing medium. Finally, PP-InsPs control cell cycle progression through the S phase after the release from START in late G1, the latter known to be controlled by PKA and TORC1–Sch9 (Jorgensen et al. 2004; Jorgensen and Tyers 2004; Moreno-Torres et al. 2015). Most interestingly, a recent study implicated the vacuole for early G1 progression and confirmed the importance for TORC1–Sch9 for this checkpoint as well (Jin and Weisman 2015).

It becomes increasingly clear that the mechanisms involved in controlling pH homeostasis and nutrient signaling are intimately connected. Here, we illustrated that these reciprocal connections also apply to Pi signaling and phosphate metabolism, including the acquisition of Pi and storage as PolyPs, as well as PP-InsP biosynthesis, all being again interconnected. The link between pH homeostasis and nutrient availability is only an emerging research field and undoubtedly there are many avenues that need to be explored to fit pH homeostasis in the intricate interplay between nutrient signaling and metabolism. Nonetheless, the impact of the intertwining of nutrient signaling, the regulation of pH, and metabolic adaptations is already obvious and, as such, it holds great promise for industrial and medical applications as well.
